# The Photoprotective Protein PsbS from Green Microalga *Lobosphaera incisa*: The Amino Acid Sequence, 3D Structure and Probable pH-Sensitive Residues

**DOI:** 10.3390/ijms242015060

**Published:** 2023-10-11

**Authors:** Vasily V. Ptushenko, Dmitry D. Knorre, Elena S. Glagoleva

**Affiliations:** 1 Belozersky Institute of Physico-Chemical Biology, Lomonosov Moscow State University, 119992 Moscow, Russia; 2 Emanuel Institute of Biochemical Physics, Russian Academy of Sciences, 119334 Moscow, Russia; 3 Faculty of Computational Mathematics and Cybernetics, Lomonosov Moscow State University, 119992 Moscow, Russia; 4 Faculty of Biology, Lomonosov Moscow State University, 119991 Moscow, Russia

**Keywords:** photoprotective protein, protonation, thylakoid lumen pH, molecular dynamics (MDs) simulation, Chlorophyta

## Abstract

PsbS is one of the key photoprotective proteins, ensuring the tolerance of the photosynthetic apparatus (PSA) of a plant to abrupt changes in irradiance. Being a component of photosystem II, it provides the formation of quenching centers for excited states of chlorophyll in the photosynthetic antenna with an excess of light energy. The signal for “turning on” the photoprotective function of the protein is an excessive decrease in pH in the thylakoid lumen occurring when all the absorbed light energy (stored in the form of transmembrane proton potential) cannot be used for carbon assimilation. Hence, lumen-exposed protonatable amino acid residues that could serve as pH sensors are the essential components of PsbS-dependent photoprotection, and their pK_a_ values are necessary to describe it. Previously, calculations of the lumen-exposed protonatable residue pK_a_ values in PsbS from spinach were described in the literature. However, it has recently become clear that PsbS, although typical of higher plants and charophytes, can also provide photoprotection in green algae. Namely, the stress-induced expression of PsbS was recently shown for two green microalgae species: *Chlamydomonas reinhardtii* and *Lobosphaera incisa*. Therefore, we determined the amino acid sequence and modeled the three-dimensional structure of the PsbS from *L. incisa*, as well as calculated the pK_a_ values of its lumen-exposed protonatable residues. Despite significant differences in amino acid sequence, proteins from *L. incisa* and *Spinacia oleracea* have similar three-dimensional structures. Along with the other differences, one of the two pH-sensing glutamates in PsbS from *S. oleracea* (namely, Glu-173) has no analogue in *L. incisa* protein. Moreover, there are only four glutamate residues in the lumenal region of the *L. incisa* protein, while there are eight glutamates in *S. oleracea*. However, our calculations show that, despite the relative deficiency in protonatable residues, at least two residues of *L. incisa* PsbS can be considered probable pH sensors: Glu-87 and Lys-196.

## 1. Introduction

The photosynthetic apparatus (PSA) of a plant provides plant growth and, at the same time, serves as a potential source of reactive oxygen species (ROS), damaging the PSA. This occurs in excess light, when the rate of electron transfer reactions limits the overall light phase of photosynthesis. As a result, low-potential reduced redox cofactors accumulate in the electron transport chain, and chlorophyll (Chl) excited state lifetime in photosynthetic antennas increases. Both of them (reduced redox cofactors and excited Chl) interact with molecular oxygen to form ROS [1,2,3]. Consequently, mechanisms protecting PSA at excess light are necessary for its sustainable functioning [4]. Among the most important of them is the regulated reversible enhancement of thermal dissipation of excitation energy [5]. Since this enhancement leads to a decrease in the Chl fluorescence intensity (so-called fluorescence quenching) and is not due to the activation of photochemical charge separation, it is referred to as non-photochemical quenching (NPQ).

NPQ is typical for all the oxygenic phototrophs, including cyanobacteria, algae, and higher plants [6,7]. Several components induced under different conditions and timescales are distinguished in NPQ. The fastest and most important component of NPQ is the so-called energy-dependent quenching, qE, activated in a seconds timescale by acidification of the thylakoid lumen (which, in turn, occurs due to proton transfer into the lumen coupled with electron transport) [5]. In Streptophyta (higher plants and Chara algae), the thylakoid membrane protein PsbS, a subunit of photosystem II (PSII), is responsible for the induction of NPQ [8], as well as special forms of xanthophylls (in the vast majority of plants—zeaxanthin) [9,10]. Without PsbS, vascular plants produce a lower NPQ and suffer from photoinhibition, especially with a rapid increase in irradiance [11]. Lower NPQ leads to higher production of reactive oxygen species [12]. Even in the moss *Physcomitrella patens* possessing both photoprotective proteins, LhcsR and PsbS, the PsbS gene knockout leads to a remarkable decrease in NPQ [13].

Suggestions about how PsbS performs its photoprotective function have changed significantly over time. Initially, it was assumed to serve as a direct quenching center due to the associated carotenoid molecules (zeaxanthin) [14,15]. But later evidence was obtained that PsbS does not bind any pigments [16,17]. All the doubts were resolved in 2015 with the help of X-ray diffraction data, which showed that the PsbS structure excludes the binding of Chl or carotenoids [18]. This led to hypotheses implying PsbS-mediated emergence of quenching centers in photosynthetic antennas through their detaching from reaction centers and subsequent aggregation into low-fluorescent complexes [19]. The pH-dependent activation of PsbS is considered to be due to its dimer-to-monomer conversion since the discovery of the reversible light-induced changes in monomer/dimer ratio (the monomer form being favored at acidic pH) [20]. This conversion is accompanied by a change in the PsbS location within the PSII supercomplex, namely, the reassociation of monomers with light-harvesting antennas, while dimers tend to associate with the PSII cores. In turn, the dimer/monomer transitions were recently suggested to be related with the changes in two short amphipathic helices, H2 and H3, at the lumenal side of the protein. In *Physcomitrella patens* PsbS, the NMR and IR spectroscopic data revealed repositioning of H2 from the aqueous to the membrane phase and switching H3 from a loop segment into a helix caused by protonation of key glutamate residues [21].

However, regardless of the mechanism of PsbS-dependent quenching, switching PsbS to the active state in response to lumen acidification remains the key process. This switching is obviously caused by the protonation of some lumen-exposed amino acid (AA) residues. While qE dependence on intrathylakoid pH has been numerously documented in the literature (e.g., [22]), proving the role of protonation of specific residues in qE induction is a much more difficult task. The search for such residues is essential for understanding the structural and functional relationships in the protein and is carried out by various methods, including methods of directed mutagenesis [21,23]. For the vascular plant *Arabidopsis thaliana* and the moss *Physcomitrella patens*, a set of mutants with replaced glutamate residues was obtained. In both species, two lumen-exposed glutamate residues were especially critical for the function of PsbS. When the three-dimensional structure of spinach PsbS became accessible, it also became possible to calculate the pK_a_ values of all assumed pH-sensing groups [24].

As mentioned above, PsbS is typical for higher plants and Chara algae. In all other groups of algae, the same function (albeit through a completely different mechanism, namely, direct quenching [25,26]) is performed by another protein belonging to the photosynthetic antenna protein superfamily, LhcSR [27,28]; it also functions in Chara algae and mosses. However, a few years ago, the first data appeared, indicating that the photoprotective function of PsbS may be more ancient. In 2016, two groups of researchers simultaneously detected short-term (in an hour timescale) stress-induced expression of the PsbS-encoding gene and its product in the cells of the green alga *Chlamydomonas reinhardtii* [29,30]. Recently, we discovered the long-term (in a day timescale) stress-induced expression of the PsbS-encoding gene in the cells of another green alga *Lobosphaera incisa* [31], which may indicate the comprehensive participation of this protein (along with the “legitimate” LhcSR) in photoprotection in Chlorophyta. One may expect that the molecular environment and operating conditions of PsbS in an algal cell differ from those in a higher plant cell. This raises the question of its properties and, in particular, the pK_a_ values of its AA residues, which determine the lumenal pH range for NPQ activation. In this regard, we searched for transcripts encoding this protein in *L. incisa*, read its presumed amino acid sequence, simulated its three-dimensional structure and calculated the pK_a_ values of its probable pH-sensing residues. In this article, we present the results of these calculations, as well as data on the amino acid sequence and the three-dimensional structure of the protein. We compare them with the data known to date on other organisms and analyze them with regard to structure versus function relations.

## 2. Results

### 2.1. The Exon–Intron Structure of the PsbS Gene

**Figure 1:**
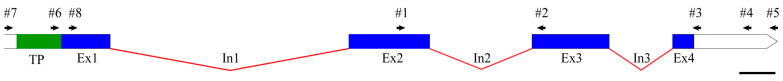
Exon–intron structure of PsbS gene. The transit peptide (TP) encoding region, exons (Ex#) and introns (In#) are marked in green, blue, and red, respectively. The arrows indicate the positions of primer #1–#8 used in the work (see Table 1). The scale bar is 100 bp.

The CDS of the gene encoding PsbS (GenBank accession number OR604507; the sequence is also presented in Supplementary Materials Table S1) is 756 bp in length. The gene contains at least four exons (the limited sequencing data do not allow us to resolve the presence (or absence) of an intron near the 5′-end of the gene). The exon–intron structure of the gene is shown in Figure 1.

### 2.2. Homology between PsbS from L. incisa and PsbS from Other Organisms

**Figure 2:**
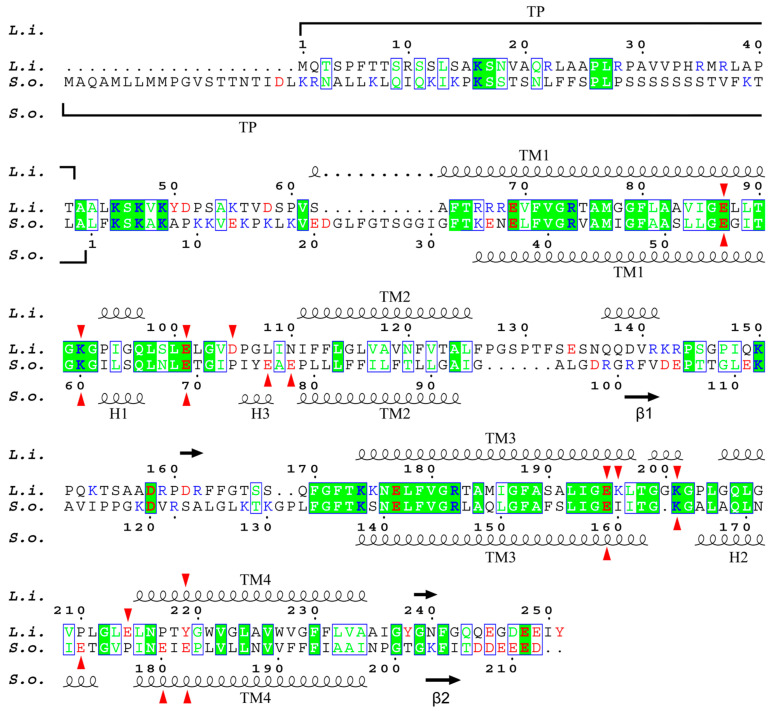
Alignment of *L. incisa*–PsbS (marked as *L.i.*) and *S. oleracea*–PsbS (*S.o.*) mature protein sequences. For *S. oleracea*–PsbS, the conventional numbering of AA residues is given (starting from the first residue of mature protein); for *L. incisa*–PsbS, numbering includes predicted transit peptide (TP). Highly conservative sites are marked with a green background; green letters indicate AA residues similar in physico-chemical properties. Protonatable residues are marked in red (anionogenic AA: Asp, Glu, and Tyr) or blue (cationogenic AA: Lys and Arg). Red arrows mark lumen-exposed glutamate residues of both proteins, as well as aspartate and lysine residues of *L. incisa*–PsbS. The secondary structure, including α-helices (wavy lines) and β-sheets (arrows), of both proteins is also shown.

Figure S1 shows the multiple alignment of the sequence of PsbS proteins from *L. incisa* and four other species of green algae belonging to three different classes (*Botryococcus braunii*, Trebouxiophyceae; *C. reinhardtii* and *Haematococcus lacustris*, Chlorophyceae; *Micromonas pusilla*, Mamiellophyceae), as well as from a higher plant *S. oleracea*. There are 19 highly conservative sites in the protein structure. This is significantly less than what was revealed when comparing the PsbS sequences of higher plants (including the moss *Physcomitrella patens*), which were 130 (of ca. 200) fully conserved residues, arranged in compact or even continuous groups up to 23 amino acid (AA) residues in length [18]. This probably indicates not only the evolutionary distance between higher plants and green algae but also a lower selection pressure in this group of phototrophs possessing the photoprotective protein LhcSR, an analogue of PsbS, along with the latter. The alignment of only *L. incisa* and *S. oleraca* protein sequences reveals a less than 33% identity (68 AA out of 210/212 AA). Moreover, all the 68 AAs are mostly dispersed along the gene singly or in groups of 2 or 3 AAs, and only 16 AAs are concentrated in three relatively small groups (7, 5, and 4 continuously located conservative AA residues) (Figure 2). Most of the conservative AA residues are located in the first and third transmembrane helices (TM1, TM3) and adjacent loci of the lumen-exposed loops, as well as in a compact (5 AA) conservative locus adjacent to the stromal end of the TM3. Therefore, it is not surprising that almost two-thirds of the coinciding AAs are hydrophobic or non-polar (42 AA); the others are 10 polar and 16 charged AA residues (9 cationic and 7 anionic).

### 2.3. The Three-Dimensional Structure of PsbS

To predict the spatial structure of a chloroplast protein based on its mRNA sequence, the chloroplast signal sequence at the N-terminus should be taken into account. The length of the chloroplast signal sequence varies from 13 to 146; most (>80%) chloroplast transit peptides consist of 30–80 residues [32]. For the studied protein, the length of the signal peptide was estimated at 41 AA.

**Figure 3:**
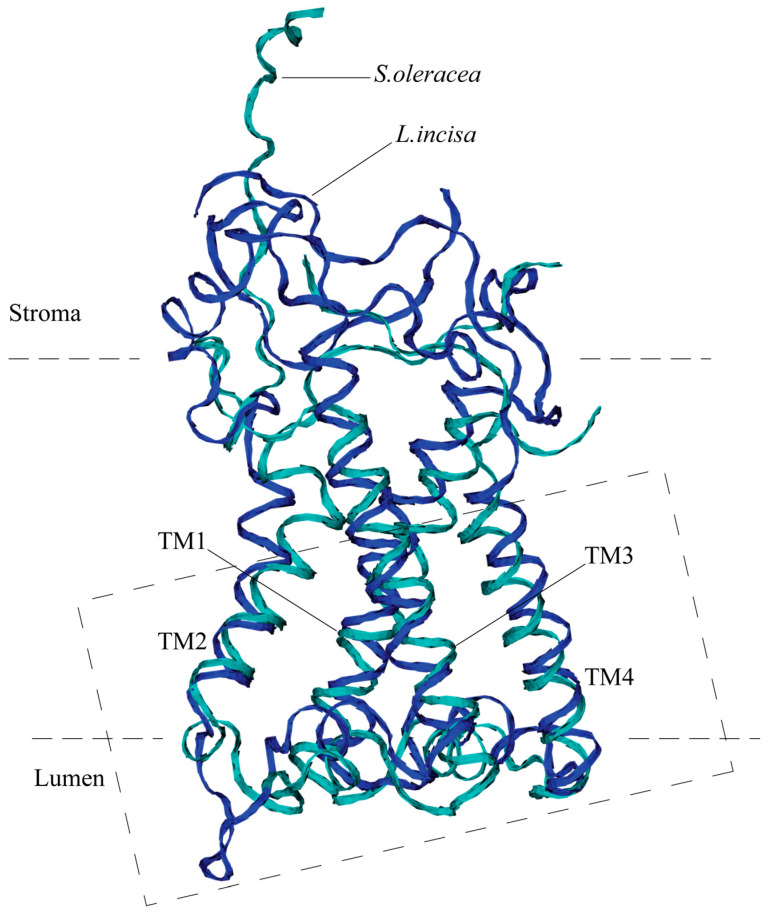
Overall 3D structure of PsbS from *L. incisa* (predicted; marked in blue) compared with that of *Spinacia oleracea* (marked in cyan). Only backbones are shown. Four transmembrane helices (TM1–TM4) are indicated. The dashed rectangle denotes the region shown in detail in Figure 4.

The predicted three-dimensional structure of the protein optimized using MD simulation is very close to that of the structure of *S. oleracea* PsbS, despite the significant difference in their amino acid sequences (Figure 3). Both proteins possess four transmembrane alpha helices, TM1–TM4. The TM1 helix (Val-61—Gly-91) of the *L. incisa*–PsbS is slightly longer (by 1–1.5 turns) than that of *S. oleracea* (Thr-33—Gly-59), and the other transmembrane helices are almost the same in length: TM2, TM3, and TM4 are built of Ile-111—Leu-125, Lys-175—Thr-198, and Leu-215—Ala-234, respectively, which corresponds to TM2–TM4 alpha helical regions in *S. oleracea*–PsbS: Pro-79—Ile-93, Ser-139—Gly-163, and Asn-179—Asn-198. The TM1 and TM2, as well as TM3 and TM4, are interconnected by relatively short sections exposed in lumens, with a short (2–2.5 turns) alpha helix located between TM3 and TM4 and approximately parallel to the membrane in both proteins. Also in both proteins, the TM2 and TM3 are connected by a large (about 50 AA) stroma-exposed loop. The loop is highly mobile, which can be concluded from the X-ray structure of the *S. oleracea*–PsbS (PDB code 4RI2) where it is not resolved. Our data on MD simulation of *L. incisa*–PsbS are consistent with this conclusion.

**Figure 4:**
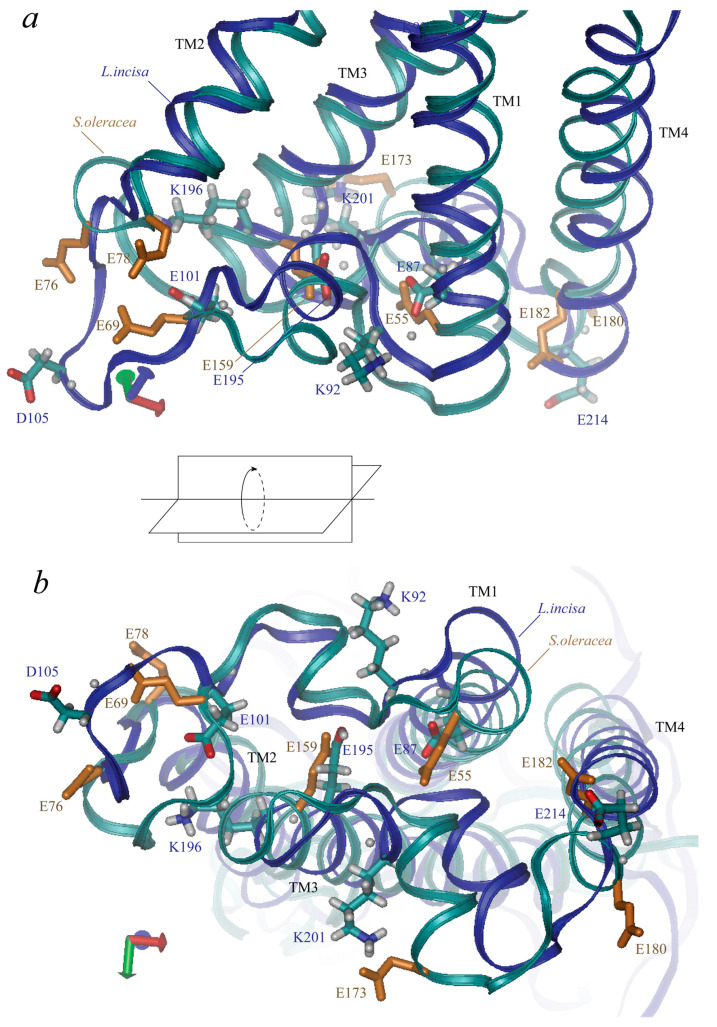
Three-dimensional structure of PsbS lumen-exposed region. Views along membrane (**a**) and from lumenal side (**b**) are presented. Along with backbones (marked in blue for *L. incisa* and in cyan for *Spinacia oleracea*), the sidechains of ionic residues are shown (in cyan-grey-red for *L. incisa* and in monotonous orange for *S. oleracea*, only glutamate residues). Four transmembrane helices (TM1–TM4) are indicated.

*L. incisa*–PsbS contains 42 ionogenic residues, including 9 Arg, 12 Lys, 4 Tyr, 10 Glu, and 7 Asp residues (here we refer to the mature protein; predicted chloroplast transit peptide contains 7 more ionogenic residues, 5 Arg, 1 Lys and 1 His). Neither His nor Cys residues, which could also be considered candidates for the role of pH sensors, are present in the mature protein. Nine of them are located at the lumenal side of the protein: glutamates 101, 195, 87, and 214, aspartate 105, tyrosine 219, lysines 92, 196, and 201 (Figure 4). Four glutamates represent two almost symmetrical pairs: Glu-87 and Glu-195 are located symmetrically at the ends of the “inner” transmembrane helices, TM1 and TM3, the shortest distance between their atoms is ca. 6–7 Å. Glu-101 and Glu-214 are located near the ends of the “outer” transmembrane helices, TM2 and TM4, the distance between them is larger than 27 Å. (Glu-101 is rather located in the lumenal loop connecting TM1 to TM2, which slightly violates the symmetry of the arrangement of the lumenal glutamate residues). Two lysine residues, Lys-92 and Lys-201, are also located symmetrically at the two lumenal loops almost between Glu-87 and Glu-195 (but 4–5 Å nearer to the surface of the protein). The other two residues, Asp-105 and Lys-196, are located closer to Glu-101 and Glu-195. The tyrosine residue, Tyr-219, is located in TM4.

Residues Glu-87, -101, -195, and -214 in PsbS from *L. incisa* correspond to residues Glu-55, -69 (one of the two pH-sensing glutamates), -159, and -180 in PsbS from *S. oleracea* (Figure 4). Glu-173 from *S. oleracea*–PsbS, which is one of the two pH-sensing glutamates, has no analogue in *L. incisa*–PsbS; it is approximately symmetric to *L. incisa*–Asp-105. Note that there are no aspartates at the lumen-exposed domains of the *S. oleracea*–PsbS. *L. incisa*–Lys-92 and Lys–201 correspond to *S. oleracea*–Lys-60 and Lys–164. *L. incisa*–Lys-196 and Tyr-219 have no analogues in *S. oleracea*–PsbS.

### 2.4. Spatial Distribution of Dielectric Permittivity in the PsbS Protein

**Figure 5:**
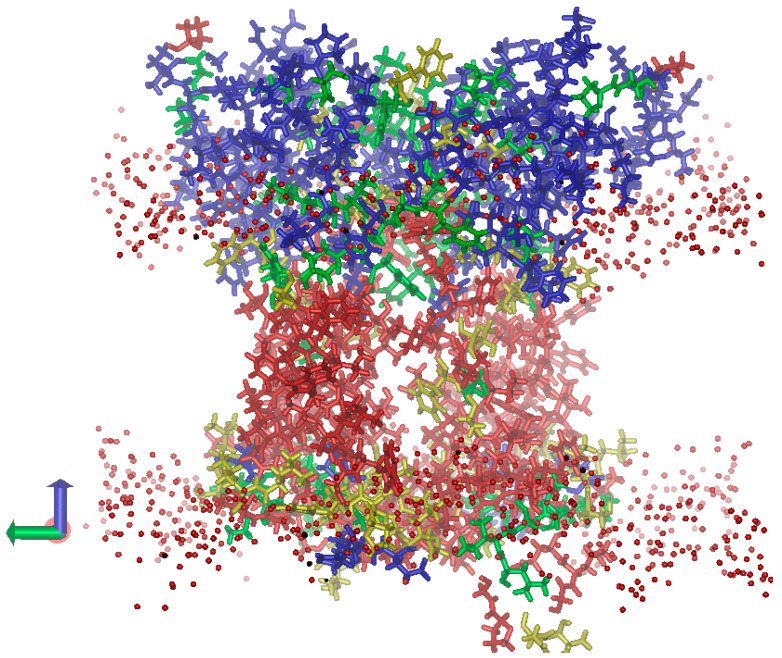
Distribution of dielectric permittivity (*ε*) within the *L. incisa*-PsbS volume (the view along the lipid membrane). The PsbS dimer submerged into the lipid membrane is shown. The protein regions with the lowest *ε* (around 3.0) are shown in red; the more polar regions with *ε* values around 5, 10, and 20 are shown in yellow, green, and blue, respectively. The oxygen atoms of polar heads of membrane lipids are shown as red spheres.

As expected, inside the hydrophobic part of the membrane, mostly hydrophobic AA residues are located, which leads to the lowest dielectric permittivity (*ε*) of this protein region (Figure 5). In the regions surrounded by lipid polar head groups, the density of polar AA residues (and hence *ε*) is much higher. The stroma-exposed region of the protein is much larger than the lumen-exposed one; therefore, the contribution of polar (including ionic) AA residues to the overall *ε* of this protein region is more significant. This results in a high *ε* (~20) of the stroma-exposed region. On the contrary, hydrophilic loops connecting the transmembrane helices in the lumen-exposed region are relatively small, and even the eight ionic residues mentioned above cannot provide a significant up-shift in the *ε* values. We assume that such a design of the dielectric properties of the protein is not accidental; rather, it is important for the functions of the protein. The low *ε* of the lumen-exposed region is a prerequisite to providing (i) strong local electric fields and (ii) high values of the dielectric response of the medium; both factors ensure a larger shift in the pK_a_ of the lumen-exposed ionic AA residues toward their biologically relevant values (i.e., closer to the neutral or slightly acidic pH range).

### 2.5. pK_a_ Values of the Lumen-Exposed Amino Acid Residues of PsbS

According to our calculations, there is a slight asymmetry in the pK_a_ values of ionic AA residues between the two chains of the PsbS homodimer emerging in the course of MD simulation. We revealed two residues belonging to one of the chains with pK_a_ values falling into the biologically relevant range of 5.0–7.0: Glu-87 (pK_a_ 6.75 ± 0.03) and Lys-196 (pK_a_ 5.81 ± 0.02). As for Lys-196, in both chains, we observed its sidechain somewhat submerged in the protein, which should contribute to discharging of the residue, i.e., to the down-shift in its pK_a_ value toward the neutral pH range. This seems to indicate its physiological role as a pH sensor. However, in one of the chains, we observed a permanently protonated state of Lys-196 stabilized by hydrogen bonds (H-bonds) formed by hydrogen atoms of its terminal amino group with carbonyl oxygen atoms of the backbone (namely of Glu-101, Leu-102, and Gly-103). As for Glu-87, in the other chain it also revealed a higher pK_a_ value compared with the other glutamates, although it was insufficient to provide its physiological role (~5.0).

On the contrary, Glu-195 in both chains forms an H-bond (as an H-bond donor, i.e., employing a carboxyl H atom), which stabilizes its protonated state and up-shifts pK_a_ toward the physiologically irrelevant alkaline range. At least in one of the chains, we observed an H-bond formed by a Glu-195 carboxyl proton with one of the carboxyl oxygen atoms of Glu-101. This, in turn, stabilizes Glu-101 in the deprotonated state in the neutral and slightly acidic pH region. Nevertheless, in the other chain, Glu-101 did not form this H-bond and manifested a pK_a_ value close to 5.0.

As for Glu-214 and the aspartate residue Asp-105, they are located almost on the surface of the protein, so their pK_a_ shifts due to the dielectric response energy should be negligible. Moreover, in one of the chains, we observed an additional stabilization of the deprotonated state of Asp-105 due to two H-bonds formed by its carboxyl oxygen atoms with the backbone hydrogen atom of Gly-107 and one of the hydrogen atoms of Pro-106. All this makes Glu-214 and Asp-105 unlikely candidates for the role of physiologically significant pH sensors.

As for tyrosine and two other lysine residues (Lys-92 and -201), there do not seem to be any effects able to downshift their pK_a_ significantly. Thus, Tyr-219 is submerged into the protein, which causes a slight stabilization of its neutral (protonated) form. Moreover, the hydroxyl proton forms (at least part of the time) a hydrogen bond with the backbone oxygen of Leu-88, which ensures additional stabilization of the protonated Tyr-219.

It is noteworthy that in the PsbS from *S. oleracea*, the lumen-exposed protonatable (presumably glutamate) residues tend to form a much more remarkable pH-sensitive cluster compared with that of *L. incisa*’s PsbS, as was shown for the first time by [24]. Their data, as well as our own obtained from calculations with the PsbS from *S. oleracea*, reveal four glutamate residues (Glu-78, Glu-173, Glu-180, and Glu-182) with the pK_a_ values upshifted above 6.0. We obtained pK_a_ values for this cluster of 6.2, 6.3, 6.4, and 7.2, which are close to those calculated by [24] (6.1, 6.3, 6.4, and 7.7). The other lumen-exposed glutamates manifest pK_a_ values below 6.0, the highest of them being 5.1 for Glu-76 and 4.7 for Glu-69 (or 5.7 and 5.2, respectively, according to [24].

### 2.6. The Errors Occurring in the Model

To draw reliable conclusions regarding the calculated pK_a_ values, we should estimate all the errors occurring in our model. Errors determined by Monte Carlo simulations are negligible (less than 0.002–0.003 pH units), and an error of a graphical assessment of pK_a_ does not exceed ±0.03 pH units. To assess the error coming from the uncertainty of parameters in our model, we varied some parameters and calculated the corresponding pK_a_ values. In most of the calculations, the membrane was considered to be dielectrically inhomogeneous, with *ε* = 2.0 for its inner region containing nonpolar fatty acid residues and *ε* = 20.0 for the outer regions containing hydrophilic heads. We also tested *ε* = 40.0 for the outer regions, which is close to the dielectric permittivity of glycerol [33]. This changed the pK_a_ values of two lumen-exposed residues by 0.06–0.08 pH units; for most of the lumen-exposed residues, the changes were in the region of 0.01–0.02 pH units. The effect of taking ε = 2.5 instead of 2.0 for the inner membrane region was less than 0.02–0.03 pH units for all these residues. The variations in the ionic strength of the solution in the range of 0.05–0.2 M exerted an effect of the same order of magnitude.

It is reasonable to assume that the most significant contribution to the error in determining the pK_a_ values should be made by fluctuations in the atomic structure of the protein. To account for this contribution, we calculated the fluctuations in the two most significant components determining the pK_a_ shift in the protein: the change in the dielectric response energy and the effect of the pre-existing electric field created by partial charges of neighboring atoms (presumably by the atoms of the backbone), excluding the atoms of protonatable groups. The third component, i.e., determined by the dielectric interaction between the protonatable groups, only accounted for pairs interacting with energy that was not less than 0.5 pH units. Usually, each ionogenic residue interacted significantly with no more than two neighbors; for most pairs, the interaction energy was relatively small, not exceeding 0.1 pH units. All the components were calculated along the last 200 ns section of the MD trajectory with a 1 ns step. The sum of all the components varied with a standard deviation (SD) ranging from 0.38 pH units for Glu-214 to 2.2 pH units for Glu-195. This is not surprising since Glu-214 is the most water-exposed surface residue; hence, it is influenced by the protein environment to the least extent. A similar situation takes place in the case of the other most “external” residue, Asp-105 (SD = 0.52). Conversely, the residue Glu-195 interacts with the protein most strongly, mainly due to the H-bonds formed with the surrounding. The fluctuations in this large value are also large. The median value of the SD of fluctuations determining the pK_a_ shift of all the lumen-exposed ionic residues is 0.85.

Note, however, that strictly speaking, these variations cannot be considered pK_a_ variations since proton exchange occurs rather slowly compared to the structure fluctuation times studied here. Thus, typical site-specific protonation and deprotonation rate constants, *k*_on_ and *k*_off_, vary in the ranges (0.6–300) × 10^9^ M^−1^ s^−1^ (which corresponds to the microsecond to millisecond protonation time scale at pH 6.0) and (0.1–3) × 10^6^ s^−1^ (microsecond deprotonation time scale), respectively [34]. Therefore, it is the averaged fluctuations of the mentioned electric interaction of the residue with a protein media that could give an adequate estimate of the pK_a_ value.

Taking into account 200 ns fluctuations of the electric interaction-induced pK_a_ shift, we obtained pK_a_ ≈ 6.5 ± 0.1 for Glu-87 and pK_a_ ≈ 6.3 ± 0.15 for Lys-196.

## 3. Discussion

The PsbS protein was previously considered typical for the evolutionary branch of higher plants (including Chara algae). Recent studies, however, significantly lengthen its history. Initially, the PsbS gene was found in the Chlorophyta genome [35,36,37]. Only several years ago, two groups of researchers simultaneously discovered the stress-induced expression of this gene, as well as its product, in the green alga *C. reinhardtii* [29,30]. The short duration of this expression served as the basis for the conclusion about the limited protective function of PsbS in the PSA of green algae, namely, limited to the very early phase of response to environmental stress factors and/or an auxiliary role in LhcSR-dependent photoprotective response. Our recent studies [31] demonstrate that the increased expression of PsbS (up to thousands-fold increase in mRNA content compared with unstressed cells) can be retained for several days after applying stress-inducing treatment; moreover, this is accompanied by a significant increase in non-photochemical quenching (NPQ) of chlorophyll fluorescence. This suggests an essential role of PsbS in the induction of NPQ during the entire period of exposure of a plant to stress factor(s). In turn, the latter implies strict requirements for the range of intrathylakoid (i.e., lumenal) threshold pH values leading to NPQ activation. Therefore, the evaluation of this range is essential for studying the protective role of PsbS in chlorophyta.

Our data show that there is no significant difference in the pH sensitivity between *L. incisa*–PsbS and its most studied homologue from *S. oleracea*, a higher plant. This indicates the functional meaning of this degree of lumen acidification, which is crucial for NPQ induction even in such distant photosynthetic organisms. On the other hand, the AA sequence of the *L. incisa*–PsbS is strikingly different from that of *S. oleracea*–PsbS and even of the PsbS from the other green algal species; moreover, this difference is observed along the entire polypeptide chain, including the regions with lumen-exposed ionic residues essential for PsbS functioning. e.g., *L. incisa*–PsbS contains half as many glutamate residues in the lumenal region of the protein as *S. oleracea*–PsbS (4 instead of 8); moreover, it lacks one of the two pH-sensing glutamate residues (i.e., the most essential for the NPQ induction) typical for both *S. oleracea*–PsbS (Glu-173) and *A. thaliana*–PsbS (Glu-226). Although there is an additional aspartate residue (Asp-105) in *L. incisa*–PsbS, it does not save the situation since it is located in a loop strongly protruding into the aqueous phase (this makes a noticeable protein-dependent pK_a_ shift impossible). Perhaps that is why Lys-196 (which does not have an equivalent lysine residue in *S. oleracea*–PsbS) turns out to be essential in pH sensing. Its location is deep enough inside the protein; moreover, near the anionic residues Glu-195 and Glu-101 is a good prerequisite for a significant pK_a_ shift toward a neutral or slightly acidic region. This comparison of two proteins is a good example of how functional properties rather than structure can be conserved during evolution.

In our study, we considered (homo)dimer of PsbS; however, we revealed a certain asymmetry: the calculation gave different (in some cases—substantially different) pK_a_ values of the same residues in two monomers. Note that we obtained pK_a_ values that fit into the physiologically important range only for one of the monomers. We realize that this may be due to an insufficient simulation time. However, on the other hand, recognition of the significance of simulation time can be interpreted as the existence of several relatively long-lived states of a protein that differ in their pH sensitivity. We believe that this assumption is in line with modern ideas about how the functional properties of proteins are related to their thermal fluctuations; e.g., fluorescence intermittency (blinking) originating from fluctuations around several discrete protein states of photosynthetic antenna and underlying the environmental control over light-harvesting/thermal dissipation is a vivid example of this relation [38].

Despite the reasonable range of pK_a_ values obtained for the PsbS protein in the literature to date [24], as well as in the present work, the question remains about the physiological meaning of the values obtained and their relationship with the process of switching PsbS to a quenching state in vivo. It is well known that zeaxanthin is one of the key participants of NPQ [9,39] along with PsbS, and it can directly quench the electronic excitation of chlorophyll molecules participating in photophysical processes in the antenna [26,40]. However, in some classical works, it has been shown that the maximal NPQ value (more precisely, the value of the energy-dependent quenching, i.e., the NPQ component qE) does not depend on the presence of zeaxanthin; instead, the intrathylakoid pH range corresponding to NPQ induction is down-shifted ([22,41], and the references therein). If this notion is correct, then it is not entirely clear how to interpret the data on pK_a_ values of PsbS residues obtained in model systems, including those resulting from MD simulation. One possible interpretation may be the conclusion that such data represent an artifact, while some other residues play a role in switching PsbS to the quenching state in vivo—e.g., those with pK_a_ values shifting to the physiologically significant range upon contact with Zea and/or components of antenna. However, this assumption does not seem to agree well with the data on mutagenesis of residues Glu122 and Glu226 in *A. thaliana*–PsbS [23] equivalent to Glu69 and Glu173 in *S. oleracea*–PsbS [18]. Another possible interpretation may suggest the flexibility of the photoprotective (including pH sensing) function of PsbS which could employ different ionic AA residues under different environmental conditions.

Nevertheless, the study of the pH-dependent properties of the PsbS protein even in such model systems makes a lot of sense since only a comparison of theoretical and experimental data, both model and in vivo, can make it possible to find out the structural and functional relationships in this protein and in the entire photoprotective system of a plant.

## 4. Materials and Methods

### 4.1. Strain, Cultivation Conditions, and Experimental Design

Soil microalga *Lobosphaera incisa* IPPAS C-2047 (Trebouxiophyceae, Chlorophyta) was cultivated as described earlier [42]. Preliminary low-coverage genome sequencing data revealed fragments of *L. incisa* DNA homologous to the *psbS* gene of vascular plants (the contig containing these fragments is deposited to GenBank, accession number OR604506; the sequence is also presented in Supplementary Materials Table S2). To obtain the CDS of the gene and amino acid sequence of its product, we isolated total RNA from the microalgal cells subjected to stress conditions, synthesized cDNA, amplified and sequenced cDNA of the presumed *psbS*. The details of these procedures are described below.

### 4.2. RNA Isolation

Total RNA was extracted from the cell sample using RNeasy Mini kit (Qiagen, Hilden, Germany). Cell pellet frozen in liquid nitrogen (~100 mg) was homogenized in a porcelain mortar. Plant RNA Isolation Aid (Thermo Fisher Scientific, Waltham, MA, USA) was added to the homogenate along with RLT lysis buffer, then clarified by centrifugation (10,000× *g*, 2 min) and RNA was isolated according to the manufacturer’s protocol. Single-stranded cDNA was synthesized using MMLV RT kit with Mint reverse transcriptase (Evrogen, Moscow, Russia) and random or Oligo(dT)_15_ primers according to the manufacturer’s protocol; 3200 ng of RNA was used for cDNA synthesis. The obtained cDNA was stored at −20 °C.

### 4.3. Primers Design

To amplify cDNA corresponding to PsbS mRNA, we used a set of primers (Table 1). Two of them (Psbs-fwd and Psbs-rev, primers for sense- and antisense-strand DNA synthesis, respectively) were designed earlier and successfully used for RT-qPCR [31]. The other seven primers were designed in such a way to obtain the longest possible fragments of cDNA. To do this, a contig presumably containing the PsbS gene was analyzed with online software for protein prediction, Augustus, http://augustus.gobics.de/submission (accessed on 28 January 2023) [43]. Several probable variants of the protein coding sequence (CDS) were predicted (*Chlamydomonas reinhardtii* or *Volvox carteri*, the only available green algal species, were indicated as target organisms). Four start codons closest to the 5′-end and three stop codons closest to the 3′-end were chosen. The sequence ending with a start codon was used to design a 5′-end primer (Table 1, primers ##6–9). The reverse-complement sequence to the one starting from a stop codon was used to design a 3′-end primer (Table 1, Primers ##3–5). The length of each primer was chosen so that the annealing temperatures of all primers (including previously used Psbs-fwd and Psbs-rev primers; Table 1, Primers ##1–2; [31]) were approximately the same (62.0–63.5 °C). Annealing temperatures were estimated using an online calculator provided by Thermo Fisher, https://www.thermofisher.com/ru/ru/home/brands/thermo-scientific/molecular-biology/molecular-biology-learning-center/molecular-biology-resource-library/thermo-scientific-web-tools/tm-calculator.html (accessed on 15 February 2023). Subsequently, Psbs-fwd (#1) with one of the 3′-end primers (##3–5) was used in PCR to amplify the 3′-end of cDNA (all the three variants were tested). To amplify the 5′-end of cDNA, Psbs-rev (#2) with one of the 5′-end primers (##6–9) was used in PCR. Based on the CDS prediction, the probable lengths of PCR products were predicted for each primer pair (Table 1).

### 4.4. cDNA Amplification and Sequencing, Gene Prediction

Both cDNA samples generated from an RNA template with random or Oligo(dT)_15_ primers were used in PCR. The former was used in PCR with each of seven primer pairs (#1 + #(3 to 5) and #2 + #(6 to 9)). The latter was additionally used in PCR with #1+ Oligo(dT)_15_ primer pair. The analysis of PCR products obtained with each of the primer pairs (except for #1 + Oligo(dT)15, #2 + 8, and #2 + #9) using agarose gel electrophoresis revealed the only band corresponding to the predicted length. Products with the maximum length (i.e., obtained with #1 + #5 and #2 + #7 primer pairs) were Sanger sequenced using Evrogen facilities (Evrogen, Moscow, Russia). These two sequences corresponded to two semi-halves of the gene (including some fragments of 5′- and 3′-UTR) and had a common section of 146 bp length; they were combined into one sequence with a total length of 1024 bp (presumably containing the entire sequence of PsbS gene) by alignment along the common section. In the combined sequence, the location of CDS was predicted using Augustus online software (see Section 4.3).

### 4.5. Sequence Alignment

For global alignment, ClustalW Multiple Alignment, implemented in Sequence Alignment Editor BioEdit 7.2.5 [44], was used. For local alignment, the local BLAST software BLAST 2.8.1+ [45] or online software, https://blast.ncbi.nlm.nih.gov/Blast.cgi (accessed on 3 February 2023) was used.

The sequences of PsbS protein from the green algae were taken from GenBank, https://www.ncbi.nlm.nih.gov/genbank/ (accessed on 10 July 2023) for *Haematococcus lacustris* (ID: AUI41105.1) or Phytozome database https://phytozome-next.jgi.doe.gov/ (accessed on 10 July 2023) for *Botryococcus braunii* (ID: Bobra.139_1s0009.1), *C. reinhardtii* (ID: Cre01.g016600_4532.1), *Micromonas pusilla* (ID: MicpuC2.EuGene.0000110272), as well as for vascular plant *Spinacia oleracea* (ID: Spov3_chr5.00161).

The exon–intron structure based on the alignment of PsbS mRNA and contig GenBank OR604506 sequences is visualized using the online service http://wormweb.org/exonintron (accessed on 10 July 2023). To visualize results of alignment, online service ESPript, https://espript.ibcp.fr (accessed on 30 September 2023) was used [46].

### 4.6. Prediction of Protein Three-Dimensional Structure

First, the chloroplast signal sequence at the N-terminus was predicted using online service TargetP-2.0, https://services.healthtech.dtu.dk/service.php?TargetP-2.0 (accessed on 6 May 2023) employing machine learning methods [47]. The predicted chloroplast transit peptide consisted of 41 residues and was cleaved. For the remaining part of the amino acid sequence (residues 42-251), its spatial structure was simulated by online service Phyre2 (developed by the Structural Bioinformatics Group of the Imperial College London; http://www.sbg.bio.ic.ac.uk/~phyre2/html/page.cgi?id=index (accessed on 8 May 2023) in the “Intensive mode” based on the protein homology to known structures as well as on ab initio folding simulation [48].

The obtained structure was used for building an input structure for molecular dynamics (MD) simulation by CHARMM-GUI Simulation Input Generator https://charmm-gui.org/ (accessed on 4 June 2023) [49]. A bilayer membrane composed of phosphatidylglycerol, 1,2-oleyl-sn-glycero-3-phospho-(1′-sn-glycerol) (PG), was simulated by the Membrane Builder [50]. The protein was inserted into the membrane and the membrane-protein system was surrounded by water molecules and ions. In total, the system of 75 × 75 × 72 Å^3^ size contained a protein, 149 lipid molecules, 6486 water molecules (TIP3P model) and 195 ions (K^+^ and Cl^−^). The input files for GROMACS were generated by the special module [51].

The obtained system was then processed by the GROMACS v. 2022.2 molecular dynamics package [52]. The minimization of energy caused by the interaction of peptide with the solvent, the equilibration of the system, and subsequent 0.6 μs simulations with 2 ps step were performed using the AMBER 98 force field. The protonation state of the ionogenic residues was chosen based on electrostatic calculations of pK_a_ values (see the Section 4.7) employing the obtained 3D structure before MD simulation. After this cycle of MD simulation, new pK_a_ values were calculated, and a new 0.2 μs cycle of MD simulation was performed. This procedure was repeated three times.

After that, two identical copies of the resulting 3D structure of the PsbS polypeptide chain were aligned with two subunits of the spinach PsbS dimer (PDB code 4RI2). For the resulting dimer embedded in the lipid membrane, MD simulation was performed again for 0.2 microseconds. Thus, the total MD simulation time was 1.4 microseconds. The resulting structure was used to calculate the pK_a_ values of protonated groups of the PsbS dimer.

The MD simulation was also performed for PsbS dimer from *S. oleracea* (PDB code 4RI2) with similar parameters of the system: 75 × 75 × 87 Å^3^ cell containing 130 lipid molecules, 8863 water molecules (TIP3P model), and 188 ions (K^+^ and 30 Cl^−^).

### 4.7. Calculations of pK_a_ Values of PsbS Residues Based on the Dielectric Properties of the Protein

To calculate the pK_a_ value of each ionogenic residue in PsbS protein, we estimated its shift upon the residue transfer from water solution into the definite site in the protein within the framework of the semi-continuum dielectric model. Three main components of the transfer energy were considered: (i) the change in the dielectric response energy (analogue of the Bornian solvation energy for liquid solvents) and (ii) the effect of the pre-existing electric field created at a given site within the protein by permanent partial charges of neighboring atoms. After that, (iii) the dielectric interaction between the protonatable groups was accounted for.

In these calculations, we used the approach developed in our previous works [53,54] and based it on accounting for dielectric heterogeneity of the protein. To assess the spatial distribution of dielectric permittivity (*ε*) within a protein, we employed correlation between local concentration of polar groups in a definite protein volume, on the one hand, and the static dielectric permittivity of the same volume, on the other hand. Previously, we thoroughly investigated this correlation using a well-studied bacterial photosynthetic reaction center, a protein complex for which the experimental data on the permittivity of its various domains are available. Following the algorithm we developed [53], in the present work, we calculated the local density of the atoms of the polar side chains bearing substantial partial charges, i.e., of the oxygen and nitrogen atoms, in different points of the protein. Based on the empirical dependence between this local density and the local *ε* value determined in the work [53], we calculated the *ε* distribution and then grouped the regions with similar *ε* values into four layers, each of which was assigned an average *ε* value: 3.0, 5.0, 10.0, or 20.0. The obtained “discrete” *ε* distribution was used to calculate the change in the dielectric response energy upon residue transfer from water into protein and the dielectric interaction between the protonatable groups. Note that the obtained *ε* is static dielectric permittivity (*ε*_s_), i.e., it accounts for both the inertial and electronic parts of the medium polarization.

In electrostatic calculations, the protein was surrounded by a bilayer PG membrane in a configuration obtained in MD simulation. The membrane was considered to be dielectrically inhomogeneous. For the inner region of the membrane formed by fatty acid residues, we used *ε*_s_ = 2.0 in most of the experiments and *ε*_s_ = 2.5 while assessing the probable error resulting from variations in this parameter. For the outer regions containing hydrophilic heads, we used the experimental data obtained for glycerol [33] and starch [55]. The former gave *ε*_s_ ≈ 40.0, and the latter gave the range *ε*_s_ ≈ 26–70 for different forms of starch. In our calculations, we used *ε*_s_ ≈ 40.0 or 20.0 for this outer membrane region to assess the probable error of the dielectric model. The protein embedded in the membrane was surrounded by a continuous medium (aqueous solution) with ε = 81 and ionic strength of 0.2 M.

To calculate the electric field and dielectric response energies, the Poisson–Boltzmann equation (PBE) was solved using DelPhi [56] V. 4 Release 1.0, supporting introduction of several dielectric regions. The numerical solution of PBE was performed in a three-dimensional grid with the space interval of 0.56 or 0.50 Å and the margins of 10–17 Å. Partial charges for amino acid residues as well as the radii of atoms (actually, the Pauling van der Waals radii) were taken from the semi-empirical parameterization scheme PARSE that was adjusted to describe solvation energies [57]; the probe radius of solvent was 1.4 Å, the ionic strength of the solution was 0.2 M.

The protonation states of ionizable residues (in total, 39 residues: 9 Arg, 12 Lys, 1 Tyr, 10 Glu, and 7 Asp) were obtained by the Monte Carlo sampling method using the program Karlsberg version 1.0.1 [58,59]. The initial pK_a_ values used as input data for Karlsberg were calculated as the sum of two components of the transfer energy of a residue: the shifts caused by the change in the dielectric response energy (the sign depends on the charge of protonated residue) and by the intraprotein electric field.

## 5. Conclusions

PsbS is a universal photoprotective protein among higher plants. Recent studies (including our own) have shown that the history of its functioning in phototrophs began at least with green algae. Given the different habitat as well as the fact that LhcsR-dependent photoprotection (absent in vascular plants) is active in green algae, one can expect a significant difference in the pattern of activation of the PsbS-dependent photoprotective pathway. This difference should correlate with the structure and physico-chemical properties of the protein. Since the activation of photoprotection is pH-dependent, the appropriate molecular tools, i.e., the set of protonatable residues of a protein and their pK_a_ values, are expected to play a crucial role.

Our study revealed a significant difference between the AA sequences of *L. incisa*–PsbS and *S. oleracea*–PsbS (coincidence of less than a third of AAs dispersed along the protein), including the regions with lumen-exposed ionic residues. *L. incisa*–PsbS contains only four glutamate residues in the lumenal region of the protein (compared with eight in *S. oleracea*–PsbS). Moreover, one of the two pH-sensing glutamate residues typical for both *S. oleracea*–PsbS (Glu-173) and *A. thaliana*–PsbS (Glu-226) is absent in *L. incisa*–PsbS. Nevertheless, the overall three-dimensional structure of the protein is very close to that of its homologues. Further, two of the protonatable lumenal-exposed residues, Glu-87 and Lys-196, are predicted to possess pK_a_ values in the slightly acidic region essential for triggering PsbS into protective mode. This may indicate that the PsbS-dependent photoprotective mechanism and the conditions for switching it on do not differ significantly in green algae and higher plants. However, this issue requires future thorough research.

## Figures and Tables

**Table 1:** Primer sequences and corresponding PCR product lengths.

No	Name	Sequence	PCR Product Length (bp)	
			Psbs-fwd	Psbs-rev
1	Psbs-fwd	CACCTTCAGCGAGTCCAAC	-	146
2	Psbs-rev	ACAGCTCGTTCTTCTTGGTG	146	-
3	Psbs-3′-1	CTTGCAGTGTTTAAATCGAGCTA	365	-
4	Psbs-3′-2	GACTAAACTTGACAGCAGCTCTA	506	-
5	Psbs-3′-3	CTCACCATCTCTCCACAAACTA	577	-
6	Psbs-5′-1	GTCGTCCCACACAGGATG	-	450
7	Psbs-5′-2	CAAACGGCTACTAGAGCAATG	-	573
8	Psbs-5′-3	TGAAGAGCAAGGTCAGTACATG	-	406
9	Psbs-5′-4	CGTACAGTCCTTAGTACAGCATG	-	1130

## Data Availability

Data are available in the paper. Further material is available from the corresponding author upon request.

## Supplementary Materials

The following supporting information can be downloaded at https://www.mdpi.com/article/10.3390/ijms242015060/s1.
