# Supplementary material for: The Photoprotective Protein PsbS from Green Microalga Lobosphaera incisa: The Amino Acid Sequence, 3D Structure and Probable pH-Sensitive Residues
Source: Int J Mol Sci. 2023 Oct 11;24(20):15060. doi: 10.3390/ijms242015060 (PMC10606523; doi:10.3390/ijms242015060)
Supplement: Supplementary file 1 [file ijms-24-15060-s001.zip › ijms-2601067-supplementary.pdf]

The photoprotective protein PsbS from green microalga *Lobosphaera incisa*: the amino acid sequence, 3D structure and probable pH-sensitive residues.

Vasily V. Ptushenko[1,2,*], Dmitry D. Knorre[3], Elena S. Glagoleva[1,4]

Supplementary Materials

Table S1. The CDS of the gene encoding PsbS (GenBank accession number OR604507).

```
>L.incisa-PsbS, CDS
ATGCAGACAAGTCCGTTCACAACCAGCCGCAGCAGCCTATCGGCCAAATCAAACGTGGCC
CAGAGACTGGCAGCCCCTCTGCGACCGGCAGTCGTCCCACACAGGATGCGCCTGGCGCCG
ACCGCTGCCCTGAAGAGCAAGGTCAAGTACGACCCCAGCGCCAAGACAGTTGACAGCCCT
GTATCAGCTTTCACACGACGCCGTGAGGTTTTCGTGGCAGAACCGCTATGGGTGGCTTC
CTGGCAGCGGTGATTGGCGAGCTATTGACCGGCAAGGGCCCCATCGGCCAGCTGAGCCTG
GAGCTTGGTGTGGACCCTGGCCTCATCAACATCTTCTTCCTGGGTCTCGTCGCCGTCAAC
TTCGTGACTGCACTGTTCCCCGGCAGCCCTACCTTCAGCGAGTCCAACCAGCAGGACGTG
CGCAAGCGCCCGTCAGGCCCCATCCAGAAGCCTCAGAAGACCAGCGCAGCAGACCGACCT
GACCGCTTCTTCGGCACATCTAGCCAGTTTGGCTTCACGAAGAAGAACGAGCTGTTTGTG
GGCCGCACTGCCATGATTGGCTTCGCCTCCGCCCTCATCGGCGAGAAGCTGACAGGCGGC
AAGGGCCCCTTGGGCCAGCTCGGCGTGCCGCTCGGTTTGGAGCTCAACCCCACCTACGGA
TGGGTTGGCCTCGCAGTCTGGGTGGGCTTCTTCCTAGTCGCTGCAATTGGCTACGGCAAC
TTTGGCCAGCAGGAGGGTGATGAGGAGATCTACTAG
```

Table S2. The sequence of contig containing these fragments of the gene encoding (GenBank accession number OR604506).

```
> Li925_1
CTCCCCCACAAGGTACAAAACGATCCTTCCCTTAGCATTCATACAAGTGTAATATGCATA
CTGACAATGAAGGGTCAGCTGTTTGTACCGGTACACACAATAGGTCTGTTGTCGTTTACA
CTATTTTACCTTGTGAAGGTGGATTAGCAGTTGGTTGTGCAGTGTACAAGAAGCAATGGT
GTGGAGAAAGTGCAGAATCTGGGTTGACCAGATGTGTGAGTATGTTTGCTGAGATATGCC
ATTCTGTTGGACCCGCTTTACAGGTGCTTACAGTGTGACAACAGCAGACCACTCACTATA
GCACGGTAAGAGTGTTGACTTCCTCACAGACTACCATCTTCGTCACTCACCATCTCTCCA
CAAACTATACTCTACTGGCAATGGACACATATGACTTCGAAGCTGCATATACACAGACTA
AACTTGACAGCAGCTCTACTGACAACATCGTACATGACCGTACAAGTTGTTCGACATCAG
TTCCTCACTGTCACACTGTATACTCAGCGTCTTATAATTCAAGCTGCCTCATGGCCTCAT
GGCATCAACATTGAACCTTGCAGTGTTTAAATCGAGCTAGTAGATCTCCTCATCACCCTC
CTGCTGGCCAAAGTTGCCGTAGCCAATTGCAGCGACTGCAGTGTACACGCACGACCCAAT
AAGCGACAGCAAGTTGCAAATTGAATTCCATGACGGAAATCACTTCCGATTTCCGTCATG
GAAATCACAGACACAACCAGGCAAAGAGAAGATTCAGCGATGGGCTGCACCACCTCAAAG
CAAGCAAGCCATTCACAGCTTTCAGTCTCACCTAGGAAGAAGCCCACCCAGACTGCGAGG
CCAACCCATCCGTAGGTGGGGTTGAGCTCCAAACCGAGCGGCACGCCGAGCTGGCCCAAG
GGGCCCTTGCCGCCTGTCAGCTTCTCGCCGATGAGGGCGGAGGCGAAGCCAATCATGGCA
GTGCGGCCCACAAACAGCTCGTTCTTCTTCGTGAAGCCAAACTGGCTAGATGTGCCGAAG
AAGCGCTGCGACACAGACAGGGTTGAGTGAGAACCGCAATGAGTATGTGATTATACTGAG
CAACGGCAGTCAAGCCCTTCAACAACAAGGACCAGCACAAGACAGACTAGTATGAAGCAG
TTTGCATGTTTAAAGATACCACCAAGATATCACCAAGTAAGAGACATTACAAGCAGGTAG
CACTAACCATCAGGGCACAGCTGGCTGTAGGGCACCTGCACCCTAGCCACTGTGGACACA
TCCACAACTCGATTATGTTAGGGTTGTGCGTGACCGACCAAGTACTCACGTCAGGTCGGT
CTGCTGCGCTGGTCTTCTGAGGCTTCTGGATGGGGCCTGACGGGCGCTTGCGCACGTCCT
GCTGGTTGGACTCGCTGAAGGTAGGGCTGCCGGGGAACAGTGCAGTCACGAAGTTGACGG
CGACGAGACCCAGGAAGAAGATGTTGATGAGGCCAGGGTCCACACCAAGCTCCAGGCTCA
GCTGGCCGATGGGGCCCTTGCCGGTCAATAGCTGCAGGACAGCCAGGCCAACAGTCAGCT
TTGTGTGACAACAACACAAAGCACTGCTGGTAACTGACAGCAACTTGGTGTAACAAAAAG
GCCTATTGCTGGCTTTTCACACAAGGCTCCTGAAGGTAGCTAACTATGTTCTTCAAGGTA
AGGTTCGTAGATGAACTGCACCAGCACTGAAAAAGATTTGAGGCAAATCGAAAGCTTATA
AGCATAAATGACCAACGGTTCCAACCGCCCTTACCTCGCCAATCACCGCTGCCAGGAAGC
CACCCATAGCGGTTCTGCCCACGAAAACCTCACGGCGTCGTGTGAAAGCTGATACAGGGC
TGTCAACTGTCTTGGCGCTGGGGTCGTACTTGACCTGAGCACATCAGATCGGCAACGGTT
GTAAGTAAGCAAGCCCGTAACGTCAGGGTGCACTTGCTGGTGTGGCAAGCAGGATCCCAA
CTCGCACTGGCGACAGCTACGTCGATGGACATGCTGCAGGGTTTTGTACCTACGACCCGA
CTTCCCAACCCAGATCAAAACCCACGTCGCCAACGAACGACTCAAGTAAGCTATGGATAG
```

TGAGTTCACGCCCTGTCCACTCCTATACAAGCGGGAAGGCCGATACAGCCACGTCTTGCT
ACCTGCTTGGGCCCGCTCGAAGTACGGCATGTACTGACCTTGCTCTTCAGGGCAGCGGTC
GGCGCCAGGCGCATCCTGTGTGGGACGACTGCCGGTCGCAGAGGGGCTGCCAGTCTCTGG
GCCACGTTTGATTTGGCCGATAGGCTGCTGCGGCTGGTTGTGAACGGACTTGTCTGCATA
AGAGCCATCGTCTGCATTGCTCTAGTAGCCGTTTGATCTGTAGGTTATGTCAGTGCGTCG
AGGATGGCAGTTGGTTGTATGTATGCGTTGAGTATGTTCAGCTGGTTTGTCTGAAGGCTG
CTTGTTTTGGGGGAGACTGTCAGCGGTCTTTCAGGGGCTTCGCGCAAGGGTCAATCCCCA
AGGGTTTGCCAGGAAGTGACGTGGGCTCCTACCAGTGGTCGAAGGAGTTGTTTAGAAGGG
GTGCTGAAAATGCACGTAAATGTCGAGGTTGCGCCCAGCAGCCAGTTGAGCGGAACCTGG
CGAACTGGTTCAGCCTCTCCCAAGTACTGGGACAGTCGGTTGATGTCAACATCGCATGGG
TCTGTGCGCGATCAGGACTGCATGGCACCTGAAGGCCCCTGTTTCTGGCATGCCAAGTGG
GGCACGGGAACCAACATGCCACAGTGTATGCTTGTGGGCAGCTGGTTCTGGTGCGCCTGG
CCTGTTGATCCTGGCAGTTTGACCTGTCGGCCCCTAACAGCATGCAACGGCTGAGGGCGA
AGGGCGAACGATCCTCATTAGCCCTCAAGCACATGCTGTACTAAGGACTGTACGGACCTT
AAAGGACTGGTACGTCCCTTGCAGCATGTGTCGATCGCGTGTGATGCATGGGAAGCACGC
TTGTGATGGTGGTGGGGCGGCAGCCGGCATACCATCCGCAAGCAAGCATATGAAGTCTAT
CTTCTTAGTTCTTATAGGTTACAGCTAGAGTACTGACACACATACAACGTGTCACTAGCT
GTTTGTGTTCTGCTGGCATGCTTGCTTCGCATGGAACCCGAACGGCCCAACCCTTTTATT
GCGAGAATGCGAGCACTGCTGGGTCCAAACACATCTATAAACTTGCTGTCAATTTTACAC
ACAGAATGGAGCTAAGTCTGAACTTTTGGAGCACGACAAACCACAACCAAAACCTTGACA
TTCAATGCTCAATACTAGTCAATGCCAGCTGACAGGGCTCGGCAGCGCGAACTCATACAT
TTATTTGCCGTATACAGCCTACTTGTCATATCACTGTCAGTTAACGACGGCTGTGCACTC
TTTCAAGGACAACAAAGGCACCTTTGCCACTTGCAGAAGTGTCACACAGCCAACCGCGAT
GGCGCTAGCTGCAAATCTGCTGCCCGATGCTTGCCGATCATCCGGTGGTCATGCGATGTC
AGGGTGGAGCAAGCAAGTAGCACTGGGCTACAT

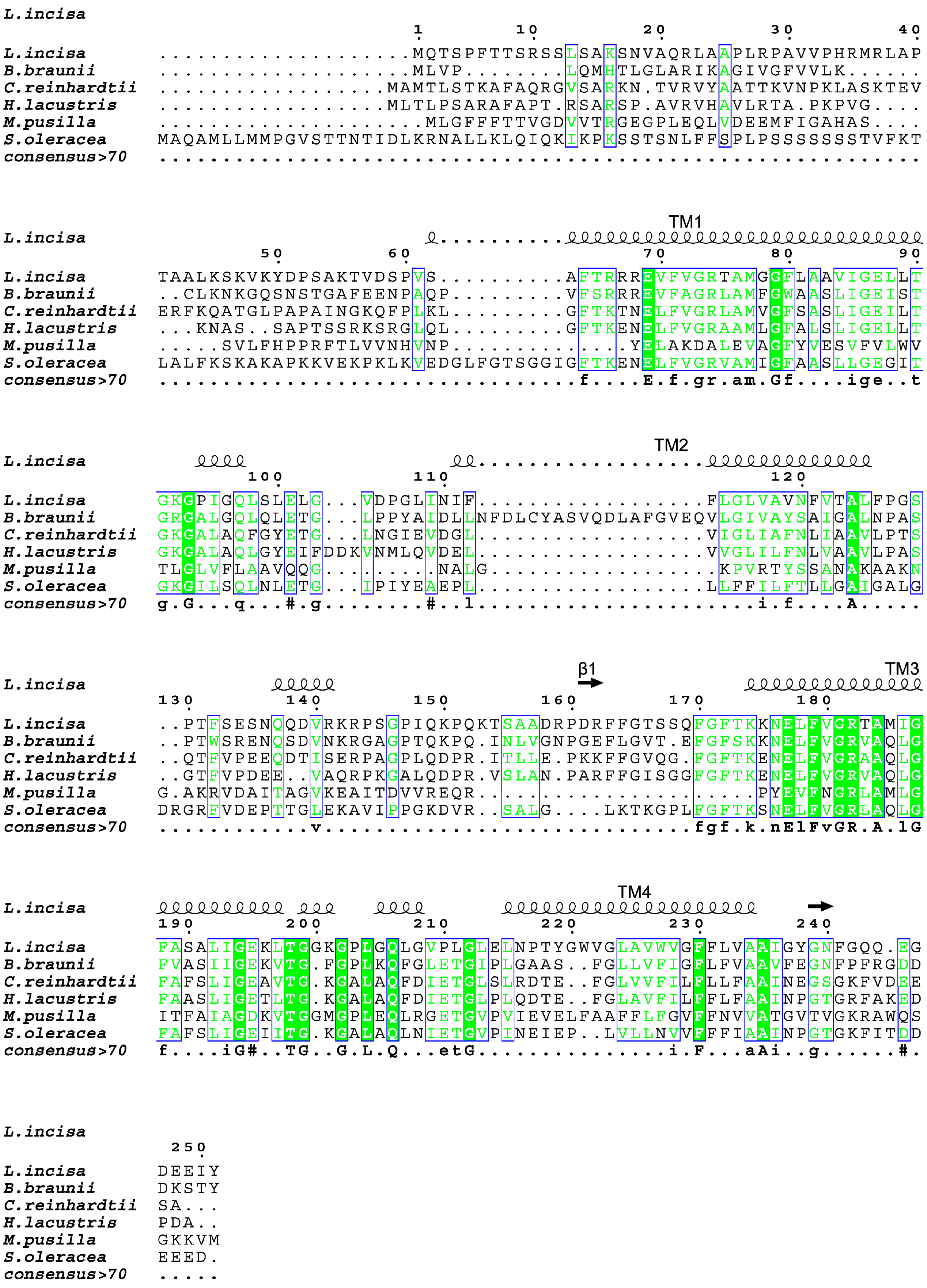

Fig. S1. Alignment of PsbS protein sequences from *L. incisa*, four other species of Chlorophyta, and a higher plant *S. oleracea*. The sequences of *L. incisa*–PsbS is predicted in this work, the other sequences are taken from GenBank (https://www.ncbi.nlm.nih.gov/genbank/) and Phytozome databases (https://phytozome-next.jgi.doe.gov/). Highly conservative sites are marked with a green background.
